# Supplementary material for: The Hippo signalling pathway coordinates organ growth and limits developmental variability by controlling dilp8 expression
Source: Nat Commun. 2016 Nov 22;7:13505. doi: 10.1038/ncomms13505 (PMC5121414; doi:10.1038/ncomms13505)
Supplement: Supplementary Information — Supplementary Figures 1-6 [file ncomms13505-s1.pdf]

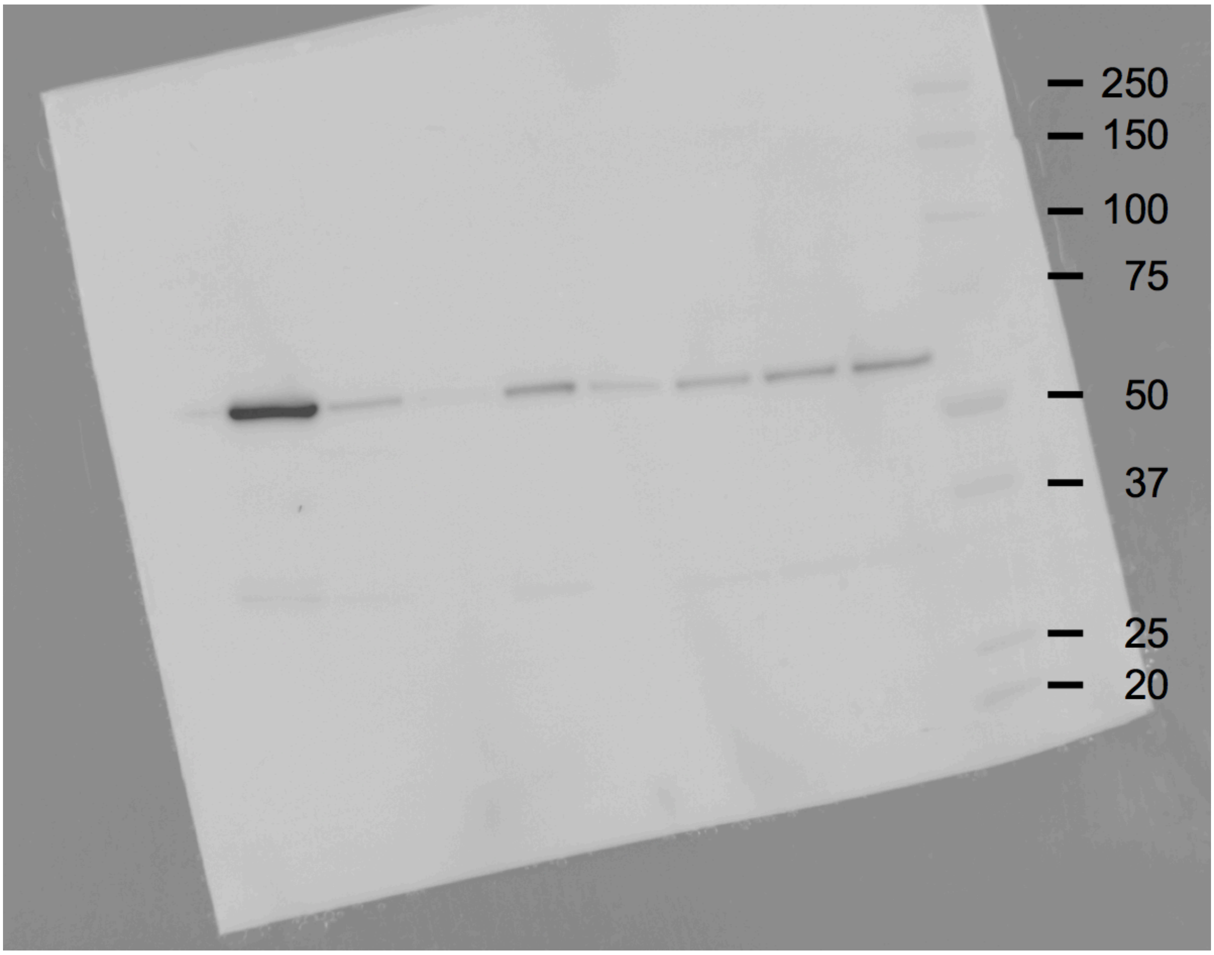

Supplementary Figure 1. Original image of a DNA pull-down experiment showing the result of anti-Flag western blotting revealing the presence of a 60KDa Flag-Scalloped band (see methods).

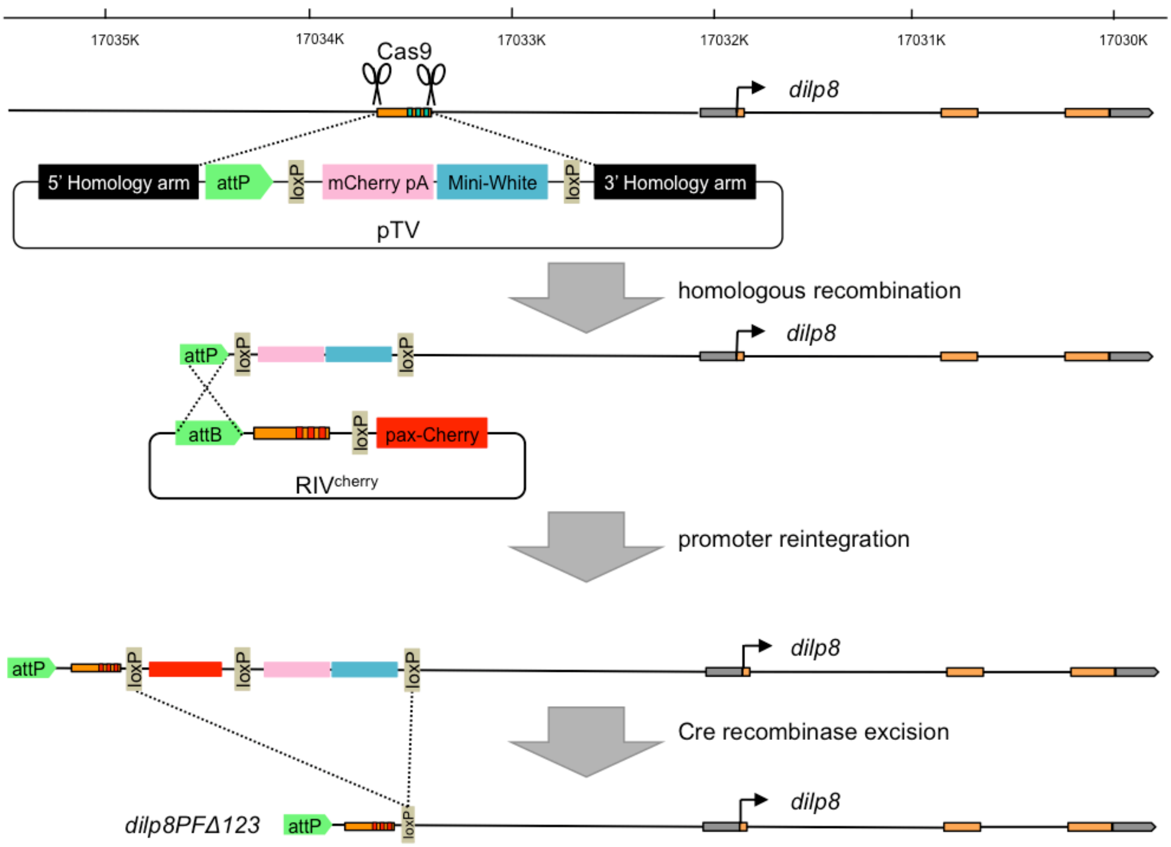

Supplementary Figure 2. *dilp8* editing by accelerated homologous recombination. Schematics illustrating the different steps employed to edit the *dilp8* promoter. The editing allows replacing a 600bp promoter fragment (PF) containing the three Sd-binding sites (orange segment with 3 green dots in between scissors) with a mutant PF containing modified Sd-binding sites (*PFΔ123*). The same procedure was also used to re-insert the *wt* promoter fragment (*PFHRE*) as a control. For a detailed description see Methods.

```
Dilp8-PF      17034117 AGGTTGTGAAATTGTATTTATGAATGAGAGTGCCCGTGGCGAACCAATTAATGCCGCATA
                       ||||||||||||||||||||||||||||||||||||||||||||||||||||||||||||
Dilp8-PFΔ123           AGGTTGTGAAATTGTATTTATGAATGAGAGTGCCCGTGGCGAACCAATTAATGCCGCATA

                       CCAGCAAGAGTTCCACCGAACCACAACGGCCAGCGCACACATTCCGAACCCAAGTCGGTG
                       |||||||||||||||||| ||||||||||||||||||||   ||||||||||||||||||
                       CCAGCAAGAGTTCCACCGTACCACAACGGCCAGCGCACAGGATCCGAACCCAAGTCGGTG

                       TTCCCAGAACGTGCCCGTTACCGTTATGCGTTGCCGATCGGATCGACGAGCGCGAGAATG
                       ||||||||||||||||||||||||||||||||||||||||||||||||||||||||||||
                       TTCCCAGAACGTGCCCGTTACCGTTATGCGTTGCCGATCGGATCGACGAGCGCGAGAATG

                       CACATTCCGAAGGATACGTGCGAAATGCGGGAGAAAATGCAACTGCGCAGCAGCATTGCG
                       |||||   |||||||||||||||||||||||||||||||||||||||| |||||||||||
                       CACATGAAGAAGGATACGTGCGAAATGCGGGAGAAAATGCAACTGCGCGGCAGCATTGCG

                       CATACGCCACGAGGGCCGGCTAACATTTCCGCATGCGGGAATGCAACGAAAGCGAAAAGC
                       |||||||||||||||||||||||||||||||||||||   ||||||||||||||||||||
                       CATACGCCACGAGGGCCGGCTAACATTTCCGCATGCGTTCATGCAACGAAAGCGAAAAGC

                       AAGTGGCAAAAAAAAAGAAAACAGCATAACCAAAACGAATGTGGAAACGAAACCGAAACC
                       ||||||||||||||||||||||||||||||||||||||||||||||||||||||||||||
                       AAGTGGCAAAAAAAAAGAAAACAGCATAACCAAAACGAATGTGGAAACGAAACCGAAACC

                       AAATCCGAACCGAATCGGAATCGGAATCAGAAACGTAACAATCTGAACCCATAAAAAGTC
                       ||||||||| ||||||||||||||||||||||||||||||||||||||||||||||||||
                       AAATCCGAATCGAATCGGAATCGGAATCAGAAACGTAACAATCTGAACCCATAAAAAGTC

                       GGCCGAAAGTGTGCATTGACCGAGAGAGCGGCTAAAAATATCCGAAAAAGAACGTAACAC
                       ||||||||||||||||||||||||||||||||||||||||||||||||||||||||||||
                       GGCCGAAAGTGTGCATTGACCGAGAGAGCGGCTAAAAATATCCGAAAAAGAACGTAACAC

                       AAACGATCTGATAAAATAAGAACGCACGATGATGACGCCCGATCCAGAACAACCGTTAAG
                       ||||||||||||||||||||||||||||||||||||||||||||||||||||||||||||
                       AAACGATCTGATAAAATAAGAACGCACGATGATGACGCCCGATCCAGAACAACCGTTAAG

                       TGCTCCACCCCAAATGATGCTGCAACAGGGTGAGCGAAAGAGAGGGCGAGCCCGATACCG
                       ||||||||||||||||||||||||||||||||||||||||||||||||||||||||||||
                       TGCTCCACCCCAAATGATGCTGCAACAGGGTGAGCGAAAGAGAGGGCGAGCCCGATACCG

                       AAAGAGACAGAGGCAGAGCCGAACA 17033517
                       |||||||||||||||||||||||||
                       AAAGAGACAGAGGCAGAGCCGAACA
```

Supplementary Figure 3. Modification of the HRE of the *dilp8* locus by gene-editing. Sequence analysis of DNA extracted from homozygous *dilp8-PFΔ123* mutants flies showing the mutations introduced by gene editing to abolish the three putative Sd-binding sites in the HRE of the *dilp8* locus (see Methods and Supplementary Fig. 1).

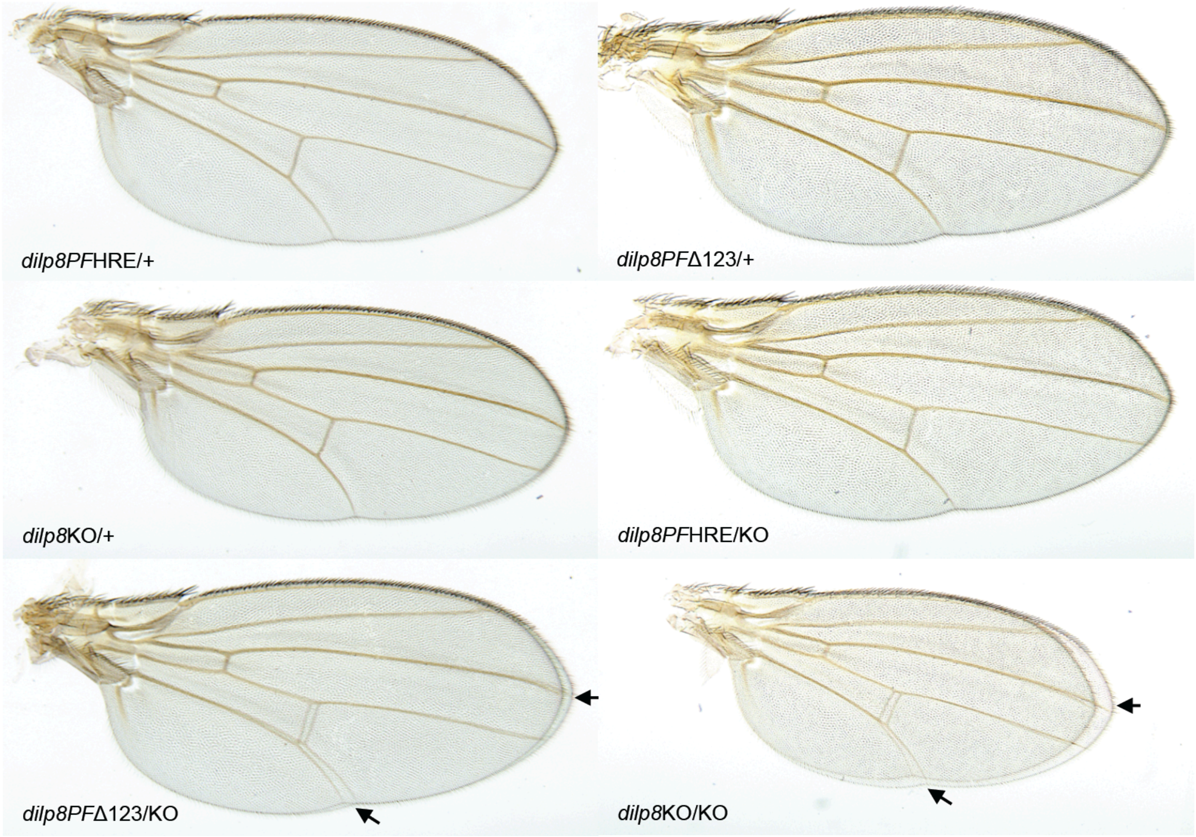

Supplementary Figure 4. Mutations in the HRE of the *dilp8* promoter induce fluctuating asymmetry. Representative pictures of overlays of left and right wings from individuals of the indicated genotypes are shown. The reduced size of *dilp8KO/KO* wings is not observed in *dilp8KO/def* mutant animals, suggesting that it is due to a secondary mutation.

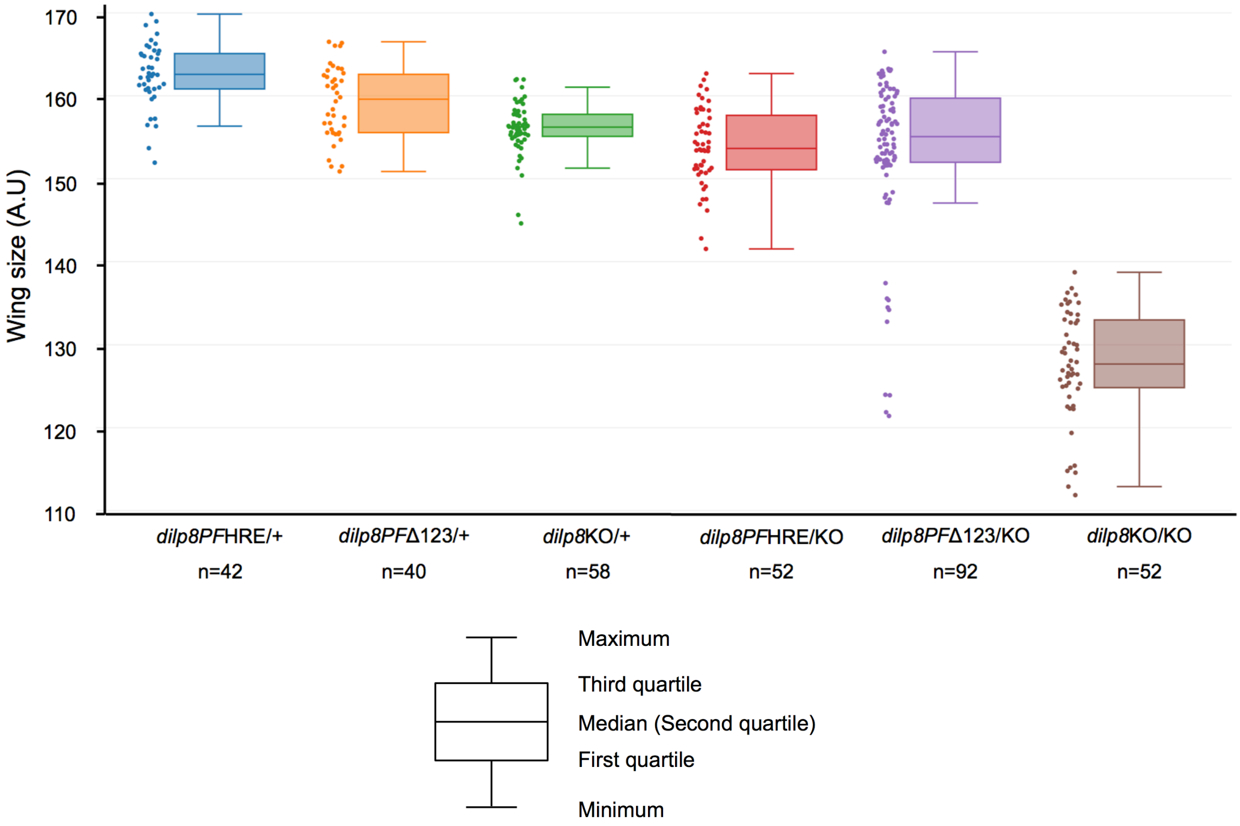

Supplementary Figure 5. Box-plot showing the distribution of wing sizes in populations of the indicated genotypes (using Plotly https://plot.ly/).

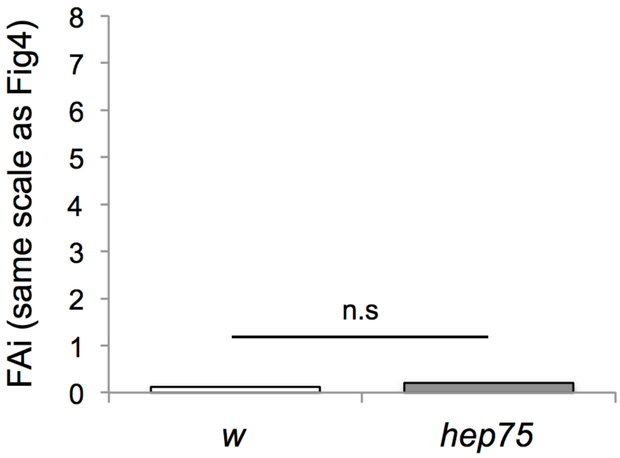

Supplementary Figure 6. Fluctuating asymmetry index measured on male individuals from w^{1118} or hep^{75} mutant background. FA index scale is as shown in Figure 4.
